# CT三维重建联合术中肺自然萎陷定位在胸腔镜肺段切除术中的初步探讨

**DOI:** 10.3779/j.issn.1009-3419.2021.101.39

**Published:** 2021-10-20

**Authors:** 肖 赵, 恒孝 卢, 振江 张

**Affiliations:** 261000 潍坊，潍坊市人民医院胸外科 Department of Thoracic Surgery, Weifang People′s Hospital, Weifang 261000, China

**Keywords:** 三维重建, 术中定位, 肺段切除, 肺磨玻璃结节, 肺肿瘤, Three-dimensional reconstruction, Intraoperative positioning, Segmental resection, Ground glass nodules, Lung neoplasms

## Abstract

**背景与目的:**

计算机断层扫描（computed tomography, CT）三维重建技术越来越多地被应用于肺磨玻璃结节（ground glass nodule, GGN）肺癌患者术前规划中，但术中如何准确定位结节和保证安全切除边缘依然是临床医生面对的难题。本研究旨在探讨全胸腔镜下肺段切除术中，CT三维重建联合术中肺自然萎陷定位方法的准确性、便捷性及切缘的安全性。

**方法:**

选取2019年7月-2019年12月收治入院的45例影像学表现有肺GGN的患者为研究组，45例患者均接受薄层CT扫描并术前进行三维重建，于麻醉后快速打开胸腔小操作口和患者气道，利用压强差使肺快速自然萎陷，根据自然标志线进行GGN定位，3-0 prolene线标记，然后行三维重建指导下的胸腔镜肺段切除术。标本摘除后测量GGN与缝线标识的距离、GGN与切缘的距离并常规送检切缘。统计患者一般临床资料、病理资料与术后并发症，并与同期采用hookwire定位针进行定位的连续45例患者进行比较。

**结果:**

CT三维重建联合术中无创式肺自然萎陷GGN定位，平均定位时间为6.9 min，定位准确率为90.6%。术中发现广泛胸腔粘连2例，肺气肿1例。术后病理均证实为肺腺癌，切缘送检均呈阴性。肺段切除后均无严重并发症的发生。

**结论:**

CT三维重建联合术中肺自然萎陷GGN定位，缩短了术中寻找GGN的时间，保障了切缘的安全性，是一种更经济、实惠、便捷的定位方法，使肺段切除更加精准。

## Abstract (en)

**Background and objective:**

Computed tomography (CT) three-dimensional reconstruction technology is increasingly used in preoperative planning of patients with ground glass nodule (GGN), but how to accurately locate the nodule and ensure the safe resection edge is still a difficult problem for clinicians. The purpose of this study was to investigate the accuracy, convenience and safety of CT three-dimensional reconstruction combined with intraoperative natural collapse localization in total thoracoscopic segmental pneumonectomy.

**Methods:**

A total of 45 patients with radiographic findings of pulmonary GGN admitted from July 2019 to December 2019 were selected as the study group. All patients received thin-slice CT scan and underwent preoperative three-dimensional reconstruction. After anesthesia, the small thoracic operation opening and the airway of the patients were quickly opened, and the lung was rapidly and naturally collapsed by pressure difference. GGN were positioned according to the natural marker line, and marked with 3-0 prolene line. After specimen removal, the distance between the GGN and the suture mark, the distance between the GGN and the incision margin were measured, and the incision margin was routinely examined. The general clinical data, pathological data and postoperative complications were counted and compared with 45 consecutive patients who were located with hookwire positioning needle in the same period.

**Results:**

The average localization time of non-invasive GGN with natural lung collapse during operation was 6.9 min, and the localization accuracy was 90.6%. There were 2 cases of extensive pleural adhesion and 1 case of emphysema. Postoperative pathology was confirmed as lung adenocarcinoma, and the examination of incision margin was negative. No GGNs were scanned again after surgery, and the precise resection rate of lung segment was 100.0%.

**Conclusion:**

CT three-dimensional reconstruction combined with GGN localization of natural lung collapse during operation can shorten the time of searching for GGN during operation and guarantee the safety of the incision margin. It is a more economical and convenient localization method and makes pulmonary segment resection more accurate.

随着高分辨计算机断层扫描（computed tomography, CT）在临床的日益普及和应用，肺癌的检出率、发病率呈现出递增趋势^[1, 2]^，特别是影像学表现呈肺磨玻璃结节（ground glass nodules, GGN）特征的早期肺癌日益增多。高度怀疑恶性的GGN仍然是以手术切除为主，胸腔镜下肺段切除（含亚肺段切除）已成为主要方式^[3, 4]^。利用CT三维重建技术，重建患者的重要肺内结构及变异，术前进行精准的靶段手术规划是实现精准肺段切除的重要一环^[5]^，但实现术前小结节的精准定位目前尚存在众多难题。例如，目前GGN定位技术较多，定位钩等多为侵入性、有创性操作，术中磁导航、B超等使用机器确定位置，操作困难且繁琐^[6, 7]^。由于肺GGN存在无实性成分、肺萎陷后不易触及等影响，主刀者仅用肉眼镜下观察和指腹牵拉触摸定位大体位置，有较大的难度和较长学习成长时间，定位不精准且困难。若无法准确确定位置，给精准肺段切除及保证安全切缘带来挑战，甚至有可能导致手术失败。本研究在术前行三维重建确定靶段手术规划的前提下，联合术中无创式肺自然萎陷定位GGN位置，达到了满意的手术切除目的，现报道如下。

## 1 资料与方法

### 1.1 一般资料

选取2019年7月-2019年12月收治入我院的45例影像学表现有肺GGN的患者（表 1），且拟行三维重建指导下胸腔镜肺段精准切除手术。对同期采用Hook-wire定位针进行定位的连续45例病例进行数据采集。本研究方案经潍坊市人民医院医学伦理委员会同意，且患者均签署了知情同意书。

**1:** 患者一般资料 General information of patients

Variables	Data
Age (Mean±SD, yr)	58.6±8.5
Gender (*n*=45)	
Male	19 (42.2%)
Female	26 (57.8%)
Location of nodules (*n*=53)	
Left upper lobe	16 (30.2%)
Left lower lobe	7 (13.2%)
Right upper lobe	18 (34.0%)
Right middle lobe	2 (3.8%)
Right lower lobe	10 (18.9%)
Smoking history (*n*=45)	
Yes	17 (37.8%)
No	28 (62.2%)
Diameter of nodules (Mean±SD, mm)	8.9±3.4
Distance from nodule to pleura (Mean±SD, mm)	12.9±4.7

### 1.2 纳入标准

① 年龄为35岁-75岁；②肺影像学呈GGN特征，直径 < 3 cm；③经过至少3个月的随访；④GGN中心位于肺野外2/3；⑤无其他恶性肿瘤或肺外转移情况。

### 1.3 排除标准

① 有明确手术禁忌证或拒绝手术者；②GGN中心位于肺野内1/3；③合并严重胸膜肥厚、胸腔积液、气胸等有可能影响肺快速萎陷的情况者；④术前无薄层CT扫描者。

### 1.4 CT三维重建术前规划

每例患者刻录DICOM格式的影像数据光盘，刻录内容为CT扫描层厚≤1 mm的薄层，动脉期、静脉期等图像。由术者应用Minics软件进行术前三维重建，重建小结节所在区域支气管及血管的走向模型，评估小结节所在肺段区域，进行术前规划。

### 1.5 定位方法

术中肺自然萎陷GGN定位需要麻醉师密切配合，待患者麻醉完成后，常规采用折刀位，消毒铺巾。在镜孔预定位置切开，同时打开患者气道，使患侧肺在大气压下快速萎陷，形成肺脏体内萎陷切迹，期间密切关注患者生命体征。待肺自然萎陷后，健侧单腔通气。于肺内寻找自然萎陷的前萎陷切迹和后萎陷切迹，留用。在二维CT横断面确定结节位置，向上计算其至肺尖、肺三角区、肺下界位置比例，左右计算其至前钝角线、后钝角线位置比例。患者健侧折刀位后，肺在大气压强下成比例自然萎陷，但其上下径和左右径位置比例关系不变。二维影像中前钝角线对应前萎陷切迹，后钝角线对应后萎陷切迹。肺组织自然萎陷后默认为一平面，应用在二维CT横断面测量数值等比例划分，确定一条垂线；同理，根据向肺尖和三角区等特殊位置的测量数值等比例划分，确定一条水平线，术中数值测量均为经高温、高压、无菌算尺测量。两线相交处即为GGN位置，用3-0 prolene线打结作标记（图 1、图 2），再辅以指腹触摸验证，记录此过程的时间。对采用Hook-wire定位针进行定位的病例，从第一次CT扫描开始，直至最终扫描确认定位钩送达满意为止结束，记录此过程用时并收集定位后并发症出现情况。

**1:**
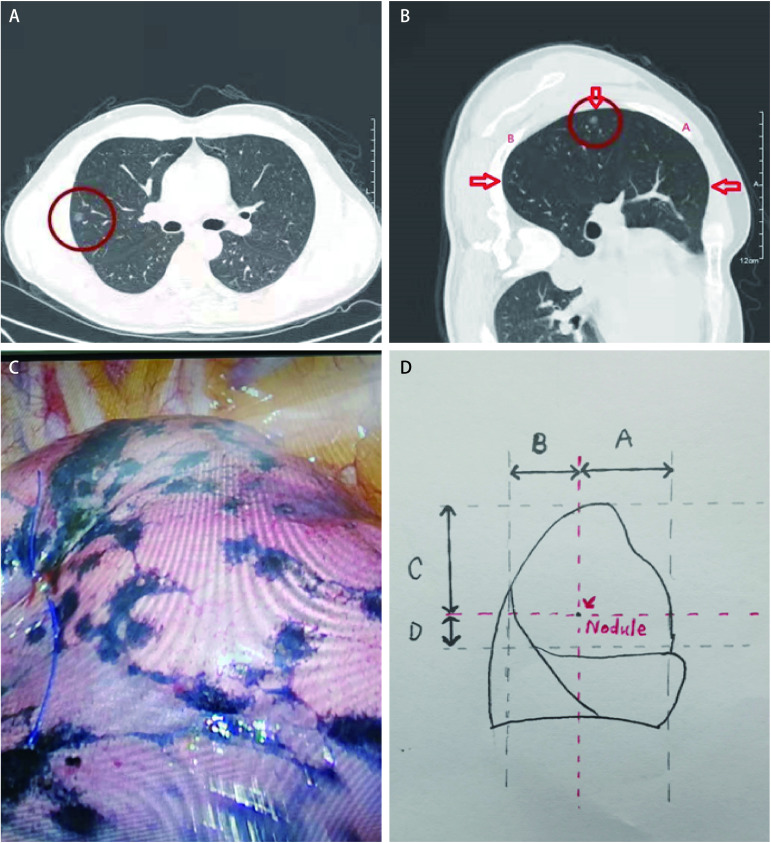
二维CT确定结节垂直比例位置。A、B：二维CT中结节所处位置比例关系；C：标记结节位置；D：等比例划线手绘图：在CT横断面确定结节位置，所示箭头处即为肺自然萎陷时的切迹点。分别测量两切迹点至结节的弧线距离A、B，计算A、B比例关系。肺萎陷后按照相同比例做一垂直线；同理，根据结节位置向上计算其至肺尖、向下计算其至肺斜裂水平裂交界处层面，计算结节所处比列位置关系，肺萎陷后按照相同比例做一水平线；两线交汇处用3-0 prolene线标记。 Two-dimensional CT to determine the vertical proportion of nodules. A, B: The proportion of nodules in 2D CT; C: Mark the location of nodules; D: Proportional drawing with lines drawn. The location of the nodules was determined in a CT cross-section, with the arrow shown as the notch point for natural lung collapse. The distance A and B from the two notch points to the curve of the nodules were measured respectively, and the proportional relationship between A and B was calculated. After the lung collapsed, a vertical line was made in the same proportion. Similarly, according to the location of the nodules, the nodules were calculated upward to the pulmonary tip and downward to the junction of the horizontal cleft of the oblique fissure of the lung, and the position relationship of the nodules was calculated. After the lung collapsed, a horizontal line was made in the same proportion. The intersection of the two lines was marked with 3-0 prolene lines. CT: computed tomography.

**2:**
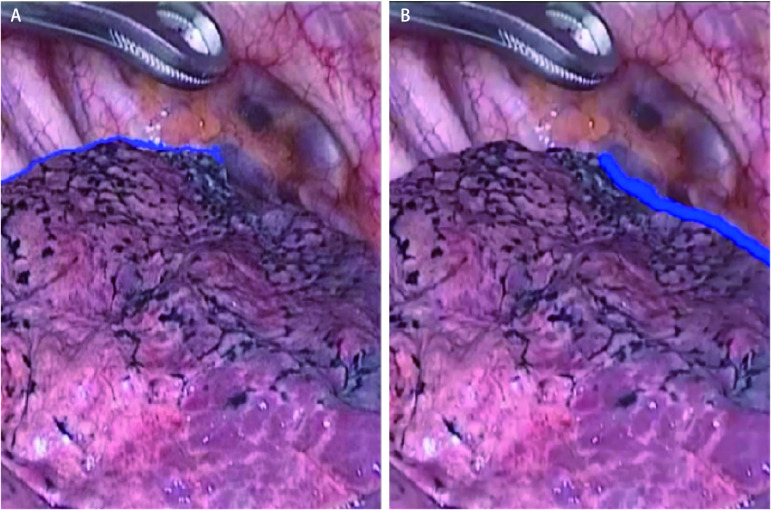
术中肺自然萎陷萎陷切迹。A：对应图 1B处自然萎陷切迹；B：对应图 1A处自然萎陷切迹。 Natural lung collapse and collapse incisions during surgery. A: Corresponds to the natural collapse notch in Figure 1B; B: Corresponds to the natural collapse notch in Figure 1A.

### 1.6 手术方法

缝线标记后，根据术前三维重建规划，仔细辨认需要切除肺段的重要结构，以直线切割闭合器或丝线结扎离断气管、血管，通气萎陷法行肺段精准切除术，切缘同时兼顾缝线标记处，距离至少2.5 cm以上。取出标本后以缝线标记为中心寻找结节，测量GGN与缝线标识的距离、GGN与切缘的距离。送术中结节及切缘行快速病理，根据快速病理结果决定下一步手术方案。

### 1.7 统计学方法

采用SPSS 26.0软件进行统计学分析，计量资料以均数±标准差描述；计数资料用例（*n*）、占比（%）描述。计量资料行*t*检验，计数资料行χ^2^检验，*P* < 0.05为差异有统计学意义。

## 2 结果

### 2.1 术中评判标准

取出标本后以缝线标记为中心寻找结节，测量GGN与缝线标识的距离、GGN与切缘的距离。认定肺萎缩状态下以缝线基部与结节水平距离≤1.0 cm为成功， > 1.0 cm为失败。结节与最近切缘的距离≥2.0 cm且快速冰冻切缘阴性为达到安全距离， < 2.0 cm或者快速冰冻切缘阳性为未达到安全距离，需要结合具体术中情况行扩大切除。记录定位时间，以开始寻找萎陷后特殊解剖位置为起点经过定位后3-0 prolene线打结作标记为终点。

### 2.2 定位相关数据

CT三维重建联合术中肺自然萎陷GGN定位研究入组45例患者。共计定位切除结节53枚，定位成功48枚结节，准确率为90.6%。平均定位时间为6.9 min（排除胸腔粘连2例），术中发现广泛胸腔粘连2例，共2枚结节，肺气肿1例，共1枚结节。缝线基部与结节水平距离结果见表 2。

**2:** 测量结果 Measurement results

Variables	Data
Horizontal distance between suture base and tubercle (cm)	
< 0.5	19 (35.8%)
0.5 < x≤1.0	29 (54.7%)
> 1.0	5 (9.4%)
Average procedural time (Mean±SD, min)	6.9±4.1

### 2.3 术后一般资料

45例患者全部行CT三维重建指导下精准肺段切除，术中快速冰冻病理均为恶性肿瘤且切缘阴性，无扩大切除病例，切除成功率为100.0%。手术过程顺利，患者均已康复出院（表 3）。

**3:** 术后病理情况 Postoperative pathology

Variables	Data
Adenocarcinoma *in situ*	6
Minimally invasive adenocarcinoma	18
Invasive carcinoma	29

### 2.4 定位效果与并发症分析

选取同期采用Hook-wire进行术前定位的患者45例，统计其一般临床资料，定位钩需要至少两次CT扫描来确定Hook-wire在理想位置，过程较繁琐，Hook-wire组总定位时间远远大于术中快速萎陷组，且有统计学差异（*P* < 0.05）。采用CT引导下定位钩定位有一定比例的并发症发生，术中快速萎陷技术无并发症出现，但受患者肺脏情况影响（如肺气肿患者）。两种定位方法在结节大小和结节距胸膜距离虽有统计学差异（*P* < 0.05），但可能需要继续扩大研究样本例数进行验证（表 4）。

**4:** 两组定位资料比较 Comparison of positioning data between the two groups

Variables	Experimental group (*n*=45)	Positioning hook group (*n*=45)	*P*
Age (Mean±SD, yr)	58.6±8.5	60.2±7.9	0.23
Diameter of nodules (Mean±SD, mm)	8.9±3.4	8.4±3.1	< 0.01
Distance from nodule to pleura (Mean±SD, mm)	12.9±4.7	14.6±3.9	0.04
Average procedural time (Mean±SD, min)	6.9±4.1	15.8±10.4	< 0.01
Complication			
Pneumothorax	0	7 (15.6%)	
Pulmonary hemorrhage	0	3 (6.7%)	
Chest pain	0	12 (26.7%)	

## 3 讨论

随着高分辨CT的出现以及人们对健康的重视，肺癌的发病率、检出率呈现增长趋势^[1]^，越来越多的肺GGN需要我们去关注。长期随访发现有相当一部分肺GGN具有恶性倾向，因此选择合适的时机进行外科干预尤为重要。目前主流的肺GGN治疗方法为胸腔镜下肺叶或部分肺叶切除术，但是由于术中术者感官偏差与辨认结节学习曲线时间长的影响，GGN精准切除仍然是业界难点。研究者通过患者薄层扫描得到影像数据后利用电脑软件进行三维重建，分析GGN所在具体肺段，制定合理的术前规划进而行精准肺段切除^[8]^。相较于传统三维重建，术前规划、术中精准辨认血管和气管行肺段切除手术，研究者在学习了先进经验后，联合术中无创式肺自然萎陷定位GGN位置可进行精准肺段切除。通过研究发现，二者联合可大大提高精准肺段切除成功率，缩短结节触摸寻找时间，缩短手术时间，更利于患者术后恢复。

目前为了解决GGN快速定位问题，出现了非常多的新技术新方法。常见的有应用带钩钢丝、弹簧圈、螺旋金属丝、亚甲蓝、医用胶或其他液体材料等在CT引导下进行定位；应用电磁导航支气管镜引导定位；近红外荧光成像定位；超声支气管镜引导定位等新技术。综合分析可发现在CT引导下定位易出现穿刺失败、气胸、血肿、脱钩等不良事件，而且多为侵入性、有创性操作，患者有时难以接受。术中磁导航、B超等使用机器辅助确定位置，存在操作繁琐、不灵活、有感染风险。术中受术者工作年限与临床经验限制，在腔镜下肉眼观察和指腹触摸寻找结节位置存在个体差异，而且学习曲线时间较长，若结节位置较深不易触及，过分牵拉肺组织容易出现术中风险^[9]^。有相关文献^[10, 11]^报道，由于GGN特有的性质及胸腔镜可探查触摸区域有限，术中定位成功率不高。总的来说，以上定位方法均有其优缺点。本研究在术前行三维重建确定靶段手术规划的前提下，联合术中无创式肺自然萎陷定位GGN位置，达到了满意的手术切除目的。

通过与麻醉师的密切配合，使肺在大气压强下自然快速萎陷，即可形成两条天然的肺脏体内萎陷切迹。肺萎陷大致分为2个阶段，第1相是肺组织本身的弹性回缩力主导的快速萎缩（胸腔粘连及气道梗阻等可能受影响）；第2相是肺毛细血管和肺泡气体交换^[12]^。利用肺等比例萎陷原则，根据二维图像中GGN与肺尖、肺三角区等的比例关系进行定位，再辅以指腹触摸验证，在三维重建指导下，以结节标记为安全中心行精准肺段切除术，取得了较好表现。本研究共纳入患者45例，共计53枚结节，按照肺萎缩状态下以缝线基部与结节水平距离≤1.0 cm为定位成功，结节与最近切缘的距离≥2.0 cm且快速冰冻切缘阴性为达到安全距离标准，全部病例均无扩大切除，切除成功率为100.0%，从一定程度上体现了两种技术联合的优势，同时尚不能排除与病例纳入标准有关。

CT三维重建技术指导，术中无创式肺自然萎陷定位方法愈发凸显的优势包括：①术前三维重建作指导，以定位缝线处为中心进行肺段切除，相互进行验证且能最大程度保留健康肺组织并保证足够的安全切缘距离；②此定位是在患者麻醉无意识状态下进行，且平均定位时间为6.9 min，缩短传统以“眼看手触”为主的GGN寻找时间；③相较于CT引导下定位，能够减轻患者心理与躯体负担；④本方法简便易学，学习曲线与学习效果明显占优势。

虽然本组按照纳入标准入组45例患者，部分患者存在2枚GGN一并切除情况，总计定位53枚结节，回顾性分析确定成功定位48枚结节，准确率为90.6%。经查阅手术记录与记录数据，肺萎缩状态下以缝线基部与结节水平距离 > 1.0 cm出现5例，其中包含2例广泛胸腔粘连病例、1例肺气肿和2例正常状态患者。对于广泛胸腔粘连和肺气肿患者，可能原因为在肺自然萎陷过程中由于粘连牵拉无法形成等比例萎陷，肺气肿由于存在无效通气腔，也可能影响等比例萎陷。另外2例可能与结节位置较深、在肺萎陷状态时存在术者视觉角度或空间垂直偏差有关。因此，此定位方法不适用于胸腔粘连、肺气肿和结节位置较深等病例。与定位钩组相比，术中快速塌陷定位法无需多次CT扫描等繁琐过程，且定位时间远远小于定位钩组时间。在有创性CT引导定位操作后，患者有一定几率出现气胸、肺内出血、胸痛等并发症，术中快速塌陷技术是在患者全麻后进行，患者无疼痛意识等相应并发症出现，但受患者肺脏情况影响（如肺气肿、胸腔粘连患者）。两种定位方法在结节大小和结节距胸膜的距离虽有统计学差异，但术者认为可能与患者接受两种定位方法意愿不同出现意愿选择偏差有关，需要继续扩大研究样本例数做进一步验证。

在CT三维重建指导下，我们应用此方法进行术中结节定位具有较高的准确率和极高的成功率，进一步印证了三维重建的准确性。联合此定位方法后，使三维重建指导下的精准肺段切除更“精准”，同时缩短了术中寻找GGN的时间，保障了切缘的安全性，是一种更经济、实惠、便捷的定位方法。本研究仅统计至2019年底，目前正在进行更大样本的后续研究。
